# Feasibility of Using Short-Segment Instrumentation and Vertebral Body Reconstruction for the Treatment of Metastatic Disease in the Thoracic Spine

**DOI:** 10.3390/jcm15187010

**Published:** 2026-09-10

**Authors:** Daniel K. Fahim, Aimen Vanood, Lee-Onn Chieng, Andrew Ragheb, Wissam El-Fallal

**Affiliations:** 1 Department of Neurosurgery, Oakland University William Beaumont School of Medicine, Rochester Hills, MI 48309, USA; 2 Department of Neurosurgery, Corewell Health William Beaumont University Hospital, Royal Oak, MI 48073, USA; 3 Atlas Spine + Brain, Royal Oak, MI 48073, USA; 4 Department of Neurology, Mayo Clinic, Phoenix, AZ 85054, USA; 5 Department of Radiology, Corewell Health William Beaumont University Hospital, Royal Oak, MI 48073, USA; 6 Advent Health Medical Group—Neurosurgery, Daytona Beach, FL 32117, USA

**Keywords:** spinal metastasis, short-segment instrumentation, posterior vertebrectomy

## Abstract

**Background/Objectives**: When performing a vertebrectomy in the thoracic spine for metastatic disease with a pathological fracture, traditionally, surgeons include two vertebral levels above and below in the instrumented fusion. We evaluated the safety and feasibility of an alternative method involving short-segment instrumentation after a posterior transpedicular thoracic vertebrectomy and reconstruction with pedicle screw fixation at only one level above and below. **Methods**: We retrospectively reviewed our series of thirty consecutive patients treated for non-junctional (T2 to T11), single-level thoracic spinal metastasis via vertebrectomy and reconstruction with posterior short-segment instrumented fusion. The primary outcome was a need for reoperation due to construct failure. The secondary outcomes included local tumor recurrence, neurological function, pain scores, operative time, estimated blood loss, post-operative wound complications, and length of stay. **Results**: All patients had a minimum Bilsky Grade of 2 and a minimum SINS (Spinal Instability Neoplastic Score) of 10. Two patients suffered perioperative complications, but there was no construct failure or need for revision surgery at any time during the follow-up period. No patients experienced worsening neurological function, and an overall improvement in pain was seen postoperatively (7.67 versus 2.77; *p* < 0.001; 95% CI: 4.28–5.52). The mean intraoperative blood loss was 605 mL (range, 50–1200 mL; SD, 314.7 mL), and the mean operative time was 260.73 min (range, 169 to 442 min; SD, 64.4 min). The average length of stay was six days. The median length of the follow-up was 24 months (range, 1–65 months; IQR, 12–42 months). Three patients in this cohort had local tumor recurrence (two of these patients suffered from a radioresistant tumor pathology). **Conclusions**: Our results support the safety and feasibility of this less invasive technique in the management of patients suffering from metastatic disease requiring single-level vertebrectomy and reconstruction in the non-junctional thoracic spine.

## 1. Introduction

Cancer is the second leading cause of death in the United States. In 2025, there were over 2 million new cases diagnosed, with over 600,000 deaths [1]. As systemic treatments and survival improve, two-thirds of patients now develop osseous metastases. Up to 10% of patients develop spinal metastases, most commonly in the thoracic spine (60–80%), followed by the lumbar spine (15–30%) and the cervical spine (<10%) [2,3].

Surgical management of spinal metastases is multifaceted [4,5]. Despite the widespread adoption of separation surgery [6,7], some patients require a vertebrectomy with direct decompressive surgery [8]. These lengthy, invasive procedures can cause significant complications [9]. A posterior approach is preferred in the thoracic spine, where a transpedicular approach can be taken to remove the vertebral body and epidural disease, avoiding the significant pain and prolonged recovery associated with a thoracotomy [10]. Standard posterior vertebrectomy involves removal of the vertebrae harboring metastasis with polymethylmethacrylate (PMMA) or expandable cage reconstruction of the vertebral body defect followed by pedicle screw fixation at least two levels above and below the level of resection [11]. Use of a minimum of two levels of instrumentation above and below a vertebrectomy and reconstruction is widely believed to be necessary due to the presumed instability that results from performing a vertebrectomy. Due to the incision and construct length, wound healing and recovery time can be significant, potentially delaying systemic treatment and postoperative radiation [12,13,14]. A review of the major publications regarding traditional long-segment instrumentation constructs and vertebrectomy in the setting of metastatic disease is compiled in Table 1.

Unlike the traditional longer-segment approach, short-segment instrumentation involves pedicle screw fixation only one level above and below the level of vertebrectomy and reconstruction. This technique has been used to treat traumatic fractures and vertebral compression fractures [30,31]. However, it is not widely accepted in treating metastasis because of the assumption that there is a greater risk of construct failure, particularly in patients receiving concomitant treatment for their cancer.

There have been successful reports of short-segment instrumentation with posterior vertebrectomy for spinal tumor removal, but these tumors were predominantly in the lumbar spine [32,33,34,35]. Short-segment fixation in the thoracic spine was reported in two patients with tumor-induced spinal deformity [36]. Additionally, combining short-segment instrumentation with percutaneous balloon-assisted kyphoplasty for thoracolumbar metastasis is promising [37]. In the setting of high-grade epidural compression, Newman et al. demonstrated that open posterolateral decompression (via laminectomy and facetectomy), as part of separation surgery, and subsequent short-segment cement-augmented fixation were safe, with a minimal complication rate pertaining to the intervention [38]. However, for cases where transpedicular vertebrectomy is indicated, there are currently no case series or large studies examining the efficacy or durability of short-segment instrumentation in such patients requiring major anterior column reconstruction for spinal metastatic disease involving the non-junctional thoracic spine (T2 to T11).

What is not currently known is whether a short segment (a single level of instrumentation) and fusion above and below a non-junctional (T2 to T11) thoracic vertebrectomy are safe and would provide adequate stability. The hypothesis of this study is that short-segment instrumentation is both feasible and safe for the treatment of patients with non-junctional thoracic metastases (without severe kyphosis/correlating with a SINS score greater than 13) that require a vertebrectomy for optimal neurological and oncological treatment of their metastatic disease. This study presents the largest cohort of patients treated with short-segment instrumentation after gross total vertebrectomy and vertebral body reconstruction for thoracic spinal metastases.

## 2. Materials and Methods

Utilizing STROBE (Strengthening the Reporting of Observational Studies in Epidemiology) [39] recommendations, we retrospectively reviewed 30 consecutive patients with single-level, solitary thoracic spinal metastatic disease between the levels of T2 and T11 who were treated with single-level gross total vertebrectomy with anterior column expandable cage reconstruction (Medtronic Altitude Cage, Medtronic, Memphis, TN, USA) and posterior short-segment instrumentation (one level of instrumentation above and below the vertebrectomy). Any patients who had undergone previous surgical interventions at these surgical levels were excluded, but patients who required interventions at other distant levels of the spine were not excluded. All patients were operated on at Corewell Health’s flagship tertiary-care referral center hospital (Corewell Health William Beaumont University Hospital) over a 7-year period by the senior author (DKF). Institutional Review Board (IRB) approval was obtained prior to data collection. As this was a retrospective chart review, a waiver of consent was granted.

Although there are many different definitions of the “durability” of a construct, in this case series, we define durability as the resilience of the construct for the remainder of the patient’s lifespan and/or follow-up—in other words, revision-free survival. Therefore, the primary outcome measure was reoperation due to symptomatic construct failure (a need for revision surgery). Secondary outcome measures included local recurrence of tumors, improvement in pain, preservation or restoration of neurological function, estimated blood loss, operative time, length of stay, and postoperative wound complications. Additional data collected included age, primary tumor, preoperative or postoperative radiation operative level, and length of follow-up. Bilsky Grade [40] and Spinal Instability Neoplastic Score (SINS) [41] were noted for all patients. SINS score was divided into three categories: stable (scores of 0–6), potentially unstable (scores of 7–12) and unstable (scores of 13–18) [40]. Pain scores were reported using the 10-point Visual Analogue Scale (VAS), corresponding to patient-reported back pain just prior to and after undergoing surgery as well as at subsequent follow-up visits. Lower-extremity muscle strength was graded using the standard British Medical Research Council Scale (0 to 5) [42].

### 2.1. Decision Making for Operative Intervention

The majority of patients were deemed appropriate for operative intervention for resection of the involved vertebral body after a multidisciplinary neuroscience tumor board review considering all aspects of the patient’s oncological disease, systemic disease burden, neurological status, radiographic findings, SINS, Bilsky Grade, and failure of previous treatment was conducted. The remaining patients represent the rare circumstances involving rapidly progressive neurological deterioration and imaging studies revealing severe cord compression in the setting of a known radioresistant primary or unknown primary, for which an urgent decision for operative intervention was made in consultation with all involved oncology treatment providers without formal tumor board review. Progression of disease in the setting of previous radiation was frequently a factor in pursuing vertebrectomy as the most appropriate treatment option. During the study timeframe, a small number of cases required a long-segment construct due to severe kyphosis that required correction or involvement of the junctional areas of the thoracic spine (cervico-thoracic or thoraco-lumbar). All these patients had an SINS score of 14 or higher. Any patient with an SINS score of 14 or higher was excluded from this study, as the severity of the instability precluded the use of a short-segment construct. This did not provide a meaningful sample size for comparison of these two treatment groups. Furthermore, during this study period, many hundreds of patients were treated with radiation (stereotactic or conventional external beam fractionated radiation), radiofrequency ablation/kyphoplasty, and/or minimally invasive separation surgery (or combinations of these treatment modalities).

This retrospective case series involved thirty consecutive patients who underwent a single-level vertebrectomy with anterior column expandable cage reconstruction and short-segment instrumentation. These thirty patients represent all of the patients treated by the senior author who met the following criteria during the study period:

1. Having an SINS score of 13 or less;
2. Having non-junctional (T2 to T11) metastatic disease warranting vertebrectomy as determined by multidisciplinary tumor board review (except for emergency situations, as delineated above).

### 2.2. Surgical Technique for Short-Segment Instrumentation

The surgical technique is provided in great detail in the Supplementary Materials. Briefly, a pair of pedicle screws were placed below and above the diseased level. Then, the laminectomy was performed along with resection of epidural tumor. Costrotranversectomy and pediculectomy were then performed to allow access to the diseased vertebral body and resection of the tumor. This technique allowed for direct decompression and circumferential decompression of the thecal sac/spinal cord while minimizing cord manipulation and eliminating retraction. Anterior column reconstruction and fusion were then achieved with an expandable cage, and the rods were placed posteriorly to secure the corrected alignment of the spine.

**Figure 1:**
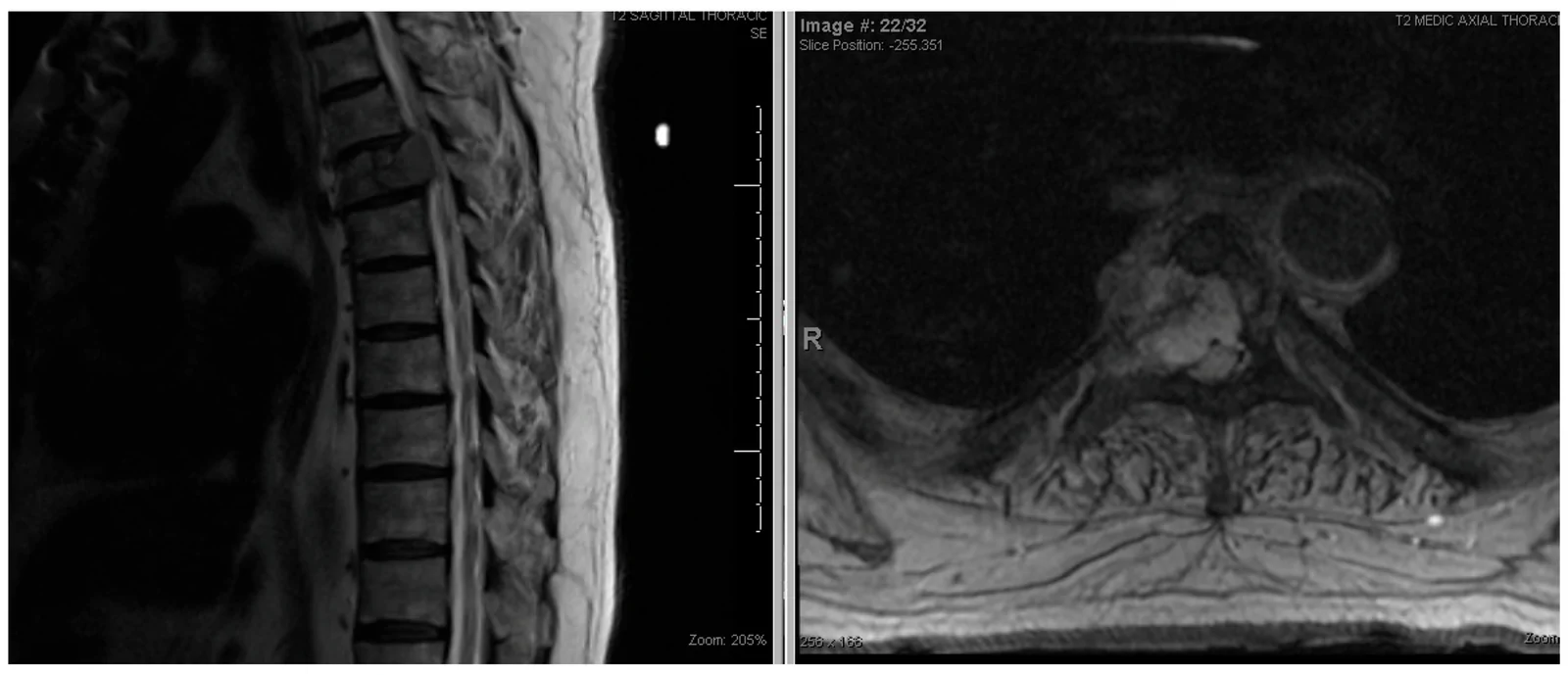
Preoperative T2-weighted MRI (A: sagittal; B: axial) demonstrating extensive T5 vertebral body metastatic lesion secondary to renal cell carcinoma with epidural extension and cord compression in a 76-year-old female.

**Figure 2:**
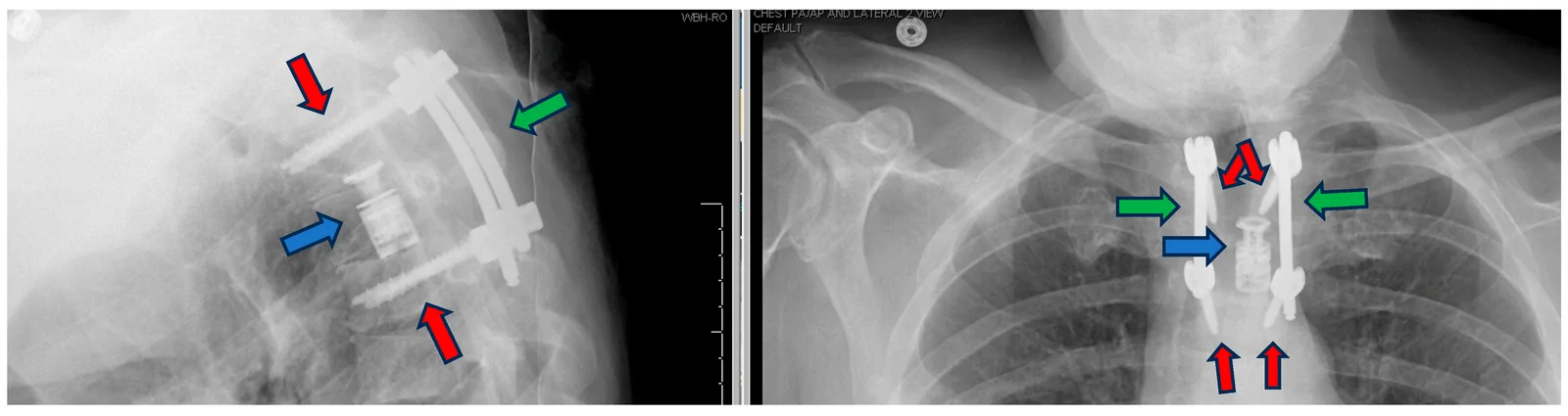
Postoperative lateral X-ray showing expandable cage vertebral body reconstruction with short-segment instrumentation from T4 to T6. The expandable cage is denoted by a blue arrow. The screws are denoted by red arrows. The rods are denoted by green arrows.

For demonstrative purposes, a preoperative MRI image and a postoperative X-ray of a 76-year-old female with metastatic renal cell carcinoma (RCC) affecting T5 (Patient 17 in Table 2 and Table 3) are shown in Figure 1 and Figure 2.

### 2.3. Follow-Up, Imaging, and Local Recurrence

Follow-ups involved a two-week post-op visit, a six-week post-op visit (which included AP and lateral X-rays), and a three-month post-op visit with an MRI of the thoracic spine with and without contrast. Subsequent imaging conducted to scan for local recurrence was performed at the discretion of the patient’s oncologist. This was usually ordered if the patient had new symptoms, positron emission tomography suggested an area of recurrence, the patient had other new sites of metastatic disease, or there was an expected increase in relevant serum markers. Additional imaging was also conducted at any time if a patient reported any new symptoms in the thoracic spine or any new neurological symptoms that could be attributed to the patient’s thoracic spine or spinal cord. Local recurrence was defined as the presence of a nodular contrast-enhancing or progressively enlarging mass lesion in the area of the previous vertebrectomy or laminectomies and tumor resection.

### 2.4. Statistical Analysis

Statistical analysis was performed using IBM SPSS Statistics version 26. Continuous variables included age, blood loss, and length of stay, which were compared using independent *t*-tests. For multiple-group comparisons (i.e., comparing tumor pathologies) of continuous or ordinal variables, Kruskal–Wallis tests were used. The categorical variables included Bilsky Grade, SINS score, muscle strength, and whether a patient received preoperative or postoperative radiation. Pain scores (VAS) were compared over time using paired-sample *t*-tests. Given the overall small sample size, categorical variables were compared using Fisher’s exact test. A *p*-value less than 0.05 was indicative of statistical significance.

## 3. Results

### 3.1. Baseline Characteristics

The average age of this patient cohort was 63.70 years (SD, 12.8 years; range, 31–84 years). The most common primary tumor was non-small-cell lung carcinoma (NSCLC), which was seen in 30% of patients (*n* = 9). Other tumor origins included prostate (23.33%, *n* = 7), renal (13.33%, *n* = 4), breast (10.00%, *n* = 3), hepatic (6.67%, *n* = 2), thyroid (6.67%, *n* = 2), chondrosarcoma metastasis (3.33%, *n* = 1), multiple myeloma (3.33%, *n* = 1), and uterine (3.33%, *n* = 1). The primary tumor origin was unknown at the time of operation for the patient suffering from multiple myeloma (patient 19). T10 and T11 were the most common spinal levels harboring metastasis (16.67% and 13.33%, respectively). Overall, 70% of patients (*n* = 21) were categorized as potentially unstable with reference to their SINS scores (range: 10–12), and the remaining nine patients had an SINS score of 13, categorizing them as unstable. With regard to Bilsky Grade, 43.30% of the cohort (*n* = 13) were Grade 2, and 56.70% (*n* = 17) were Grade 3. Radiation had been administered to 17 patients at some point prior to surgery and to 15 patients postoperatively (two patients received both preoperative and postoperative radiation). The baseline parameters are detailed in Table 2.

### 3.2. Operation and Hospital Course

The primary and secondary outcome measures are summarized in Table 3. The mean intraoperative blood loss was 605 mL (SD, 314.7 mL; range, 50–1200 mL), and the mean operating time was 260.73 min (SD, 64.4 min; range, 169–442 min). Two surgical peri-operative complications occurred. One patient had a hematoma in the setting of early postoperative systemic anticoagulation for a known history of deep venous thrombosis and pulmonary embolus. Another experienced a superficial wound infection at the operative site, which was successfully treated with antibiotics. Neither instance required reoperation. Lower-extremity muscle strength improved postoperatively in 86.67% of patients (*n* = 26) and remained unchanged for the remaining four patients. No patients experienced worsening motor strength after surgery. The average duration of postoperative hospital stay was six days (SD, 3 days; range, 4–15 days), inclusive of any inpatient rehabilitation.

### 3.3. Follow-Up and Pain Scores

The average preoperative VAS pain score was 7.67, and the average postoperative VAS pain score at the time of discharge was 2.77. A significant reduction in VAS pain score occurred postoperatively (mean difference, 4.90; 95% CI, 4.28–5.52; *p* < 0.001). The median follow-up was 24 months (range, 1–65 months; IQR, 12–42 months).). Compared to preoperative pain, the one-month (*n* = 27) and three-month (*n* = 26) pain scores were also significantly lower (with a mean of 1.56, a mean difference of 6.00, and a 95% CI of 5.24–6.76, with *p* < 0.001, at the one-month follow-up and a mean of 0.69, a mean difference of 6.81, and a 95% CI of 6.22–7.39, with *p* < 0.001, at the three-month follow-up).

### 3.4. Reoperations for Symptomatic Construct Failure and Local Recurrence

**Figure 3:**
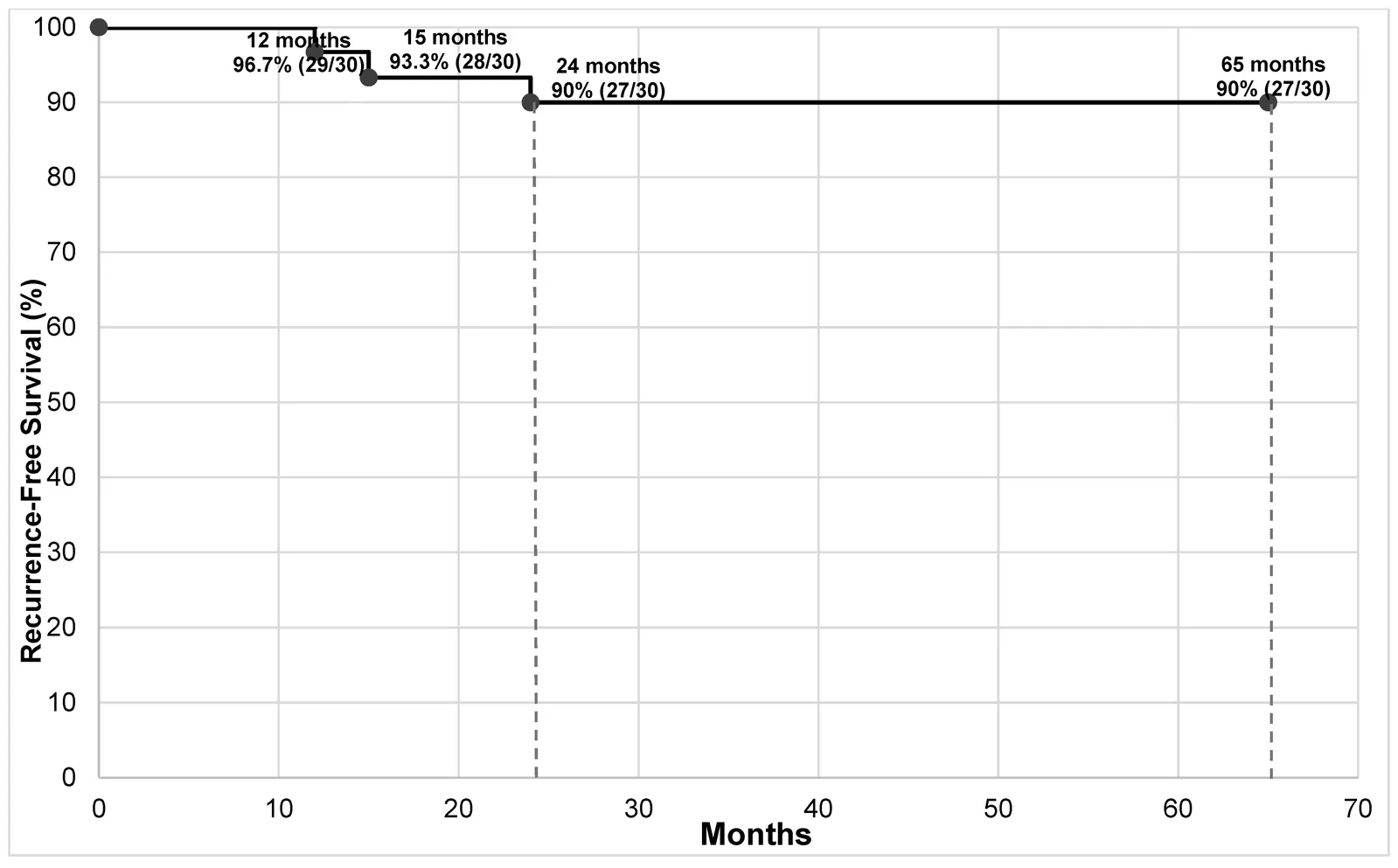
Kaplan-Meier Curve showing index spine surgery level recurrence-free survival over time among living patients.

There were no instances of construct failure or a need for revision surgery during the follow-up period. Three patients (10%) experienced local tumor recurrence. The primary tumor of origin for these three patients was renal cell carcinoma (patient 2), hepatocellular carcinoma (patient 18), and metastatic chondrosarcoma (patient 20). The Kaplan–Meier curve below shows the local recurrence-free survival as a percentage of the patients with available follow-up data (Figure 3).

### 3.5. Differences in Outcomes Based on Tumor Histology or Location

Analyses of differences in outcomes based on the histology of the tumor showed no statistically significant differences regarding neurological outcome (H = 8.80, *p* = 0.359), blood loss (H = 4.73, *p* = 0.786), or length of stay (H = 8.39, *p* = 0.397). It is essential to note that the subgroup analyses conducted according to tumor histology in this case series are exploratory and should be interpreted with caution because of the limited sample size. The small sample size likely does not provide adequate power to identify any potential differences in outcomes based on tumor histology; therefore, this comparison should only be regarded as exploratory. Similarly, no statistical differences in neurological outcomes (OR, 2.57; 95% CI, 0.24–28.09; Fisher’s exact test *p* = 0.163), EBL (mean difference, 18.3 mL; 95% CI, −204.1 to 276.0 mL; *p* = 0.878), or LOS (mean difference, 0.31 days; 95% CI, −2.00 to 2.61; *p* = 0.786) were noted when comparing patients with tumors at or above T6 to patients with tumors below T6.

## 4. Discussion

This analysis demonstrates the safety and feasibility of short-segment instrumentation after vertebrectomy for a select subset of patients with metastatic oncologic disease in the non-junctional thoracic spine (T2 to T11) with an SINS score of thirteen or less. Cancer patients are at risk of instability for a variety of reasons, which could include compromised bone quality, older age, immunocompromised state, malnutrition, and administration of radiation and chemotherapy. These factors could compromise healing and construct stability. Thus, long-segment constructs are traditionally recommended. To date, no series has examined the safety and feasibility of short-segment constructs in this highly select patient setting. However, there has been a trend towards selecting more minimally invasive techniques for the treatment of patients with metastatic disease affecting the spine [43].

In our series of thirty consecutive patients who met these criteria, there were no hardware or construct failures that required revision surgery with this short-segment instrumentation strategy. Thus, it is possible that short-segment instrumentation may be a safe and feasible option for a subset of patients who meet these criteria. In the following subsections, we discuss how the results of this case series compare to those obtained from published historical controls involving long-segment instrumentation. This only serves as a point of discussion and future scientific inquiry, as there are inherent limitations in making comparisons to historical controls.

### 4.1. Blood Loss

The mean intraoperative blood loss was 605 mL, ranging from 50 to 1200 mL. In comparison, values from reports of patients treated via long-segment instrumentation with vertebrectomy for thoracic tumor metastasis range from 400 to 2656 mL [16,18,19,20,21,24,25,26,27,28,29]. Short-segment-instrumentation patients appear to have a narrower range of operative blood loss when compared to longer-construct patients described in the literature. This is likely because the shorter-segment procedure is less invasive, exposing fewer vertebral levels. Preoperative embolization of spinal tumors is often a useful neoadjuvant adjunct for minimizing intraoperative blood loss. Bouare et al. recently described a novel embolization technique used to minimize intraoperative blood loss in renal cell carcinoma patients [44]. In this case series, the renal cell carcinoma patient did undergo preoperative embolization.

### 4.2. Operative Time

Operative time for patients in this case series ranged from 169 to 442 min. This overlaps with reported operating times for long-segment instrumentation (116 to 510 min) [16,18,19,20,21,22,23,25,26,27,28,29]. The comparable operating times, even though there were fewer operative levels, may be attributable to the fact that the majority of time was spent on tumor resection and anterior column reconstruction (identical in both surgical approaches). Thus, the operative times are comparable between the two techniques, although short-segment instrumentation appears to tend towards a shorter operative time.

### 4.3. Hospital Stay

Length of hospital stay ranged from 4 to 15 days. A similar range was seen (6–13 days) when pooling the majority of studies describing long-segment instrumentation [17,18,19,20,21,23,24,25,29]. One study, however, reported a mean length of stay of 25 days for a cohort of 17 patients treated with en-bloc vertebrectomy [15]. This study also described a higher mean operative time (782 min) and estimated blood loss (3483 mL) than other reports in the literature, which is not surprising given the greater complexity of en-bloc vertebrectomy operations.

### 4.4. Wound Infections

Although the majority of the studied patients received preoperative radiation, only two patients (6.67%) in our cohort suffered surgical perioperative complications, including one case of superficial infection at the operative site. Reported rates of complications for long-segment instrumentation vary widely in the literature. Two studies with similar sample sizes each reported one complication (2.94–3.13%) [18,19]. However, larger studies report higher rates (8.00–23.19%) [21,24]. A cohort study comparing complications for short- and long-segment instrumentation is warranted and may reveal a lower rate for patients with short-segment constructs given the shorter length of incision, which is associated with lower rates of infection and wound complications in other surgical disciplines [45,46].

### 4.5. Revision-Free Survival

Hardware failure requiring revision surgery is relatively common among patients who have undergone spinal decompression and fixation followed by radiation. This may be explained by the association between the higher risk of vertebral compression fracture and stereotactic body radiation therapy. In a study by Newman et al., no major instrumentation failures were observed, except for one patient (2%) who experienced a bilateral thoracic pedicle fracture following posterolateral decompression despite short-segment cement-augmented fixation at a mean follow-up of 10.7 months. In our study, at an average follow-up of 24 months, there were no hardware failures or vertebral fractures that required reoperation. This is not to suggest that there were no cases of radiographic screw loosening or pieces of evidence of any cage subsidence but rather that there were no symptomatic structural abnormalities that required a revision operation.

### 4.6. Pain, Neurological Outcomes, and Tumor Recurrence

Overall, patients treated with short-segment instrumentation saw a significant decrease in pain scores in the immediate postoperative period (7.67 vs. 2.77; *p* < 0.001; CI, 4.28–5.52) as well as at the one-month follow-up (7.67 vs. 1.56; *p* < 0.001; CI, 5.24–6.76) and the three-month follow-up (7.67 vs. 0.69; *p* < 0.001; CI, 6.22–7.39). The majority of patients experienced improvement in lower-extremity strength after surgery, and none had worsening motor deficits. While studies describing functional neurological outcomes in post-vertebrectomy patients are rare, Gokaslan et al. reported that only one patient out of a cohort of 72 had worsening ambulation after transthoracic vertebrectomy [47]. Thus, short-segment instrumentation appears to yield similar outcomes with regards to patient neurological function. Additionally, only three patients experienced local tumor recurrence, two of whom suffered from relatively radioresistant tumor pathologies prone to local recurrence [48,49].

### 4.7. Limitations

This preliminary feasibility study has multiple limitations. First, the short-segment-instrumentation procedures were conducted by a single neurosurgeon at a single tertiary-care referral center. Furthermore, as this was a retrospective case series, there was no control group consisting of long-segment-instrumentation patients that could be used as a point of comparison. Instead, metrics from the current literature were used to demonstrate potentially comparable safety of short-segment instrumentation in this select patient population. While this study does present the largest sample size of patients treated with short-segment instrumentation for spinal metastasis, it remains smaller than the larger cohort of standard posterior vertebrectomy patients in the pooled literature. In addition, construct durability was primarily assessed by revision-free survival rather than by systematic radiographic evaluation of implant integrity. Detailed radiological outcomes—which could be a harbinger of potential future failure of a construct outside the follow-up period—were not collected. Furthermore, the only patient-reported outcomes included were pain scores. Finally, multivariate analysis was not performed given the limited sample size. Ultimately, prospective studies are needed to further evaluate the efficacy and durability of short-segment instrumentation in the care of these complex patients.

## 5. Conclusions

Short-segment instrumentation may be a safe and feasible less invasive and promising alternative to standard long-segment constructs after a posterior transpedicular vertebrectomy and anterior column reconstruction for patients who require surgery for metastatic disease in the non-junctional thoracic spine (T2 to T11) with an SINS of 13 or less and without a significant kyphotic deformity. There were no instances of patients requiring revision surgery after an average of 24 months of follow-up. Additionally, rates of perioperative complications were low. Patient outcomes regarding pain and motor strength markedly improved, as noted in published outcomes involving longer constructs. This study supports consideration of short-segment instrumentation for this select group of patients when they require vertebrectomy. Future prospective studies comparing short-segment instrumentation to long-segment instrumentation in this patient cohort would be essential to further define the role of this more limited surgical approach.

## Figures and Tables

**Table 1:** Parameters for traditional long-segment-instrumentation cohort.

Study	Number of Patients	Average Age (yr)	Average Preop Pain Score	Operating Time (min)	Blood Loss (mL)	Number of Patients with Operative Complications	Average Postop Pain Score	Average Duration of Stay (Days)	Number of Patients with Local Relapse	Average Length of Follow-Up (Months)	Number of Patients Dying within 6 Months
Araujo et al., 2018 [15]	17	44.5	-	782.2	3483	6	-	25.4	-	-	5
Dreimann et al., 2017 [16]	14	63.6	7.2	278	2257	2	4.4	-	-	12	-
Gezercan et al., 2016 [17]	22	50.04	6.81	-	-	7	2.59	7.86	-	18.18	-
Mody et al., 2016 [18]	32	50	-	510	923	1	-	8	-	-	3
Joubert et al., 2015 [19]	34	63.1	8.94	164	862	1	2.62	8.83	-	13.7	-
Zairi et al., 2012 [20]	10	64	5.5	170	400	-	2	6	0	10.1	4
Metcalfe et al., 2012 [21]	50	55.9	-	252	1743	4	-	7.9	1	17	1
Gasbarrini et al., 2012 [22]	14	48	-	388	-	1	-	-	-	45	-
Cappuccio et al., 2011 [23]	1	56	-	120	-	0	-	10	-	30	0
Xu et al., 2009 [24]	69	55	-	-	2656	16	3.85	13	-	-	-
Jain et al., 2008 [25]	2	58	-	420	675	-	-	9	0	24	0
Crocker et al., 2008 [26]	1	65	-	235	700	0	-	-	0	-	1
Xiao et al., 2007 [27]	28	-	-	116	400	-	-	-	2	28.4	0
Muhlbauer et al., 2000 [28]	17	64.1	-	330	1595	6	-	-	1	11	6
McLain, 1998 [29]	5	-	-	435	1800	-	-	7.5	3	8	0

Summary of parameters collected from studies meeting inclusion criteria after review of the literature. Abbreviations: yr, year; preop, preoperative; min, minutes; mL, milliliters; postop, postoperative; -, data unavailable (not reported in the publication).

**Table 2:** Baseline data for short-segment instrumentation cohort.

Patient	Age (Years)	Origin of Primary Tumor	Tumor Level	SINS	Bilsky Grade	PreOp XRT	PostOp XRT	Length of Follow-Up (Months)
1	62	Renal	T2	13	3	No	Yes	12
2	31	Renal	T9	11	2	Yes	Yes	30
3	34	Renal	T9	10	2	Yes	Yes	9
4	76	Hepatic	T8	12	2	Yes	--	2
5	57	NSCLC	T6	13	3	No	Yes	2 *
6	77	NSCLC	T3	12	3	No	Yes	6
7	71	Thyroid	T11	13	2	Yes	Yes	48
8	75	Thyroid	T10	12	3	No	Yes	24
9	54	Breast	T11	11	3	Yes	No	48
10	66	Prostate	T6	10	2	Yes	Yes	28
11	66	NSCLC	T4	12	3	No	Yes	18
12	51	Uterine	T7	12	3	No	No	0.25 *
13	62	Breast	T5	12	3	Yes	Yes	42
14	72	NSCLC	T6	13	3	No	No	12
15	65	NSCLC	T6	13	2	Yes	No	65
16	79	Breast	T10	11	3	Yes	No	36
17	76	Renal	T5	10	3	Yes	Yes	23
18	80	Hepatic	T10	12	3	No	Yes	12
19	70	Multiple Myeloma *	T9	13	3	No	Yes	46
20	58	Chondrosarcoma	T11	13	2	No	Yes	18
21	59	NSCLC	T8	13	3	No	No	0.5 *
22	58	Prostate	T4	12	2	Yes	No	48
23	62	Prostate	T7	11	2	Yes	No	36
24	49	Prostate	T10	11	3	No	--	--
25	84	NSCLC	T11	13	2	No	No	3
26	67	Prostate	T8	11	3	Yes	No	36
27	61	NSCLC	T3	12	3	Yes	No	15
28	46	NSCLC	T10	11	2	Yes	Yes	39
29	68	Prostate	T3	10	2	Yes	No	36
30	75	Prostate	T5	11	2	Yes	No	12

Summary of baseline characteristics for the 30 patients. Abbreviations: NSCLC (non-small-cell lung carcinoma); SINS (Spinal Instability Neoplastic Score); PreOp (preoperative); XRT (radiation); PostOp (postoperative); -- (lost to follow-up, so data is not available); * (pathology was not known at the time of surgery).

**Table 3:** Primary and secondary outcome measures for short-segment instrumentation patients.

Patient	Reop Because of Construct Failure	Local Recurrence	PreOp Leg Strength	PostOp Leg Strength	PostOp Change in Leg Strength	Preop Pain (VAS)	Postop Pain (VAS)	One Month Pain (VAS)	Three Month Pain (VAS)	OR Time (Minutes)	EBL (mL)	LOS (Days)	PostOp Wound Complications
1	No	No	4−	4+	Improved	9	5	2	0	291	700	7	None
2	No	Yes	4+	5	Improved	7	0	0	0	286	800	4	None
3	No	No	4+	5	Improved	7	4	1	0	309	600	5	None
4	No	No	4+	4+	Same	9	2	0	--	274	500	10	None
5	No	No	4−	5	Improved	6	2	4	0	234	200	15	Hematoma
6	No	No	4−	4	Improved	8	1	0	0	378	1200	7	None
7	No	No	4	4+	Improved	9	4	0	0	442	1100	6	None
8	No	No	3	4	Improved	8	3	1	0	254	400	6	None
9	No	No	4−	5	Improved	8	4	4	1	260	600	4	None
10	No	No	4+	5	Improved	6	2	1	0	249	900	4	None
11	No	No	3	4+	Improved	7	0	0	0	185	200	9	None
12	No	No	4−	5	Improved	8	5	--	--	207	800	4	None
13	No	No	4−	4+	Improved	6	0	4	0	250	900	5	Wound infection
14	No	No	3	4	Improved	8	3	2	0	260	250	4	None
15	No	No	4+	5	Improved	9	3	2	0	251	1200	6	None
16	No	No	4	4	Same	7	0	1	1	189	500	4	None
17	No	No	4−	4	Improved	6	2	2	1	273	600	5	None
18	No	Yes	3	4−	Improved	8	1	2	2	249	400	5	None
19	No	No	3	5	Improved	9	4	0	0	322	200	11	None
20	No	Yes	4+	5	Improved	10	6	3	2	402	500	8	None
21	No	No	4−	4+	Improved	10	6	--	--	175	450	13	None
22	No	No	4+	5	Improved	9	5	1	1	228	800	4	None
23	No	No	4+	5	Improved	7	0	0	0	169	400	5	None
24	No	No	4−	4+	Improved	8	6	--	--	229	1000	4	None
25	No	No	4−	4−	Same	9	2	3	3	204	500	6	None
26	No	No	4−	4+	Improved	5	0	1	0	270	400	4	None
27	No	No	4−	4	Improved	8	2	2	2	189	50	2	None
28	No	No	4+	5	Improved	9	7	5	4	224	1000	3	None
29	No	No	4+	5	Improved	4	0	0	0	316	750	5	None
30	No	No	4+	4+	Same	6	4	1	1	253	250	9	None

Summary of outcome measures for the 30 patients. Abbreviations: ReOp (reoperation); PreOp (preoperative); PostOp (postoperative); VAS (visual analog scale); EBL (estimated blood loss); LOS (length of stay); -- (patient was lost to follow-up, so no data are available).

## Data Availability

Datasets are available to interested readers. Please contact the corresponding author.

## Supplementary Materials

The following supporting information can be downloaded at https://www.mdpi.com/article/10.3390/jcm15187010/s1, Detailed Surgical Techniques.

## Abbreviations

The following abbreviations are used in this manuscript:

PMMA	Polymethylmethacrylate
IRB	Institutional Review Board
SINS	Spinal Instability Neoplastic Score
VAS	Visual Analog Scale
CT	Computed Tomography
PLL	Posterior Longitudinal Ligament
RCC	Renal Cell Carcinoma
IBM	International Business Machines
SPSS	Statistical Package for the Social Sciences
NSCLC	Non-Small-Cell Lung Carcinoma
PreOp	Preoperative
PostOp	Postoperative
XRT	Radiation
SD	Standard Deviation
EBL	Estimate Blood Loss
ReOp	Reoperation
