# Supplementary material for: Feasibility of Using Short-Segment Instrumentation and Vertebral Body Reconstruction for the Treatment of Metastatic Disease in the Thoracic Spine
Source: J Clin Med. 2026 Sep 10;15(18):7010. doi: 10.3390/jcm15187010 (PMC13607368; doi:10.3390/jcm15187010)
Supplement: Supplementary file 1 [file jcm-15-07010-s001.zip › jcm-4465694-supplementary.pdf]

*Article*

# Feasibility of Using Short-Segment Instrumentation and Vertebral Body Reconstruction for the Treatment of Metastatic Disease in the Thoracic Spine

**Daniel K. Fahim [1,2,3,*], Aimen Vanood [4], Lee-Onn Chieng [2], Andrew Ragheb [5] and Wissam El-Fallal [6]**

## ***Supplementary Material: Detailed Surgical Technique***

The patient was placed prone on a Spinal Jackson Table. A precise skin incision was localized allowing for instrumentation at one level above and below the vertebrectomy level. After the spine was exposed, the stereotactic frame was secured to a spinous process of the upper or lower operative level and intraoperative CT scan was obtained with the O-Arm and uploaded to the Stealth Station for navigation (Medtronic; Minneapolis, Minnesota). Navigation was used to advance an undersized tap just past the posterior vertebral body wall at the level above and below the planned vertebrectomy. The superior pedicle screws were placed immediately, however, the pedicle screws at the inferior level were only placed after tumor resection was complete and anterior column reconstruction achieved. This minimized the risk of limiting access to the inferior aspect of the diseased vertebral body, disc, and posterior longitudinal ligament (PLL).

Posterior decompression was begun using rongeurs to remove the involved posterior elements. A microscope is brought into the field and generous laminectomies are performed, followed by unilateral or bilateral costotransversectomies, facetectomies, and pediculectomies, depending on the laterality of the pathology. The vertebral body was removed from one side if the disease was unilateral but approached from both sides if bilateral. Nerve root ligation was performed unilaterally to allow access for expandable cage placement.

The table was rotated away from the surgeon and the microscope angled to visualize the ventral epidural space. The PLL was resected from the level of the rostral to the caudal disc along with discectomies above and below the resected vertebral body. The endplates were gently decorticated. Spinal reconstruction in this series was performed with an expandable cage packed with allograft, to restore height after removing the fractured vertebral body and maintain or restore normal spinal alignment.

Additional allograft was added to the anterior column reconstruction for arthrodesis, followed by placement of a hemostatic agent circumferentially

around the completely decompressed thecal sac. Pedicle screws were placed in the previously tapped inferior vertebral body level. Next, rods were placed, and under gentle compression the construct was secured with set screws. The posterolateral surfacest were then decorticated and allograft for used for the supplemental posterolateral arthrodesis.
